# A combined progressive muscle relaxation and walking intervention for adults with end-stage kidney disease receiving hemodialysis (*Fight Fatigue*): Study development and protocol

**DOI:** 10.1016/j.conctc.2026.101631

**Published:** 2026-03-24

**Authors:** Mary F. Hannan, Michael J. Fischer, Victoria deMartelly, Natalie Lif, Nathan Tintle, Ardith Z. Doorenbos

**Affiliations:** a Department of Biobehavioral Nursing Science, College of Nursing, University of Illinois Chicago, Chicago, IL, USA; b Department of Medicine, College of Medicine, University of Illinois Chicago, Chicago, IL, USA; c Medical Service, Jesse Brown VA Medical Center, Chicago, IL, USA; d Center of Innovation for Complex Chronic Healthcare, Edward Hines Jr. VA Hospital, Hines, IL, USA; e Department of Population Health Nursing Science, College of Nursing, University of Illinois Chicago, Chicago, IL, USA

**Keywords:** Patient partnership board, Physical activity, Mind-body, Steps

## Abstract

**Background:**

Fatigue has a profound negative impact on health for adults with end-stage kidney disease (ESKD) who receive maintenance hemodialysis. There are currently no standard-of-care therapies to alleviate this fatigue. Progressive muscle relaxation and physical activity have decreased fatigue in other populations. In collaboration with a patient partnership board, we developed an intervention aimed at reducing fatigue for adults receiving maintenance hemodialysis (*Fight Fatigue*), and we describe the protocol for its feasibility trial.

**Methods:**

*Fight Fatigue* was informed by our formative work with a patient partnership board (n = 3). Three virtual meetings were held, followed by 1:1 interviews with each board member. During the meetings, qualitative data was collected about the board's experience with fatigue and their feedback on the components of the intervention, which focused specifically on progressive muscle relaxation and increasing steps per day, as well as the study procedures. The *Fight Fatigue* intervention was developed informed by these findings. In the planned feasibility trial of this intervention, participants will be enrolled and randomized to either the *Fight Fatigue* intervention or an attention control. Data will be collected at baseline, week 6, and week 12. For this trial, the primary study endpoints are feasibility and acceptability, with a secondary endpoint of fatigue.

**Conclusion:**

The findings of this feasibility trial will inform the development of a randomized controlled trial of a combined mind-body and physical activity intervention to address fatigue for adults with ESKD receiving hemodialysis.

**Clinicaltrials gov:**

NCT07014111.

## Highlights

• Fatigue is a prevalent and distressing symptom for those with end-stage kidney disease (ESKD).
• The *Fight Fatigue* intervention was informed by work done with a patient partnership board.
• *Fight Fatigue* will be evaluated in a 12-week trial investigating feasibility and acceptability.

## 1 Introduction

Fatigue affects up to 97% of adults with end-stage kidney disease (ESKD) receiving hemodialysis and is reported to be one of the most concerning symptoms [1,2]. Fatigue has a negative impact on morbidity, quality of life, social relationships, and functional independence in those with ESKD [[3], [4], [5], [6], [7], [8], [9], [10], [11]]. There are no standard-of-care therapies to alleviate the fatigue experienced by those with ESKD [3,4,12]. Progressive muscle relaxation, which involves the mindful tensing and releasing of muscles, has been found to relieve fatigue in chronic disease populations but has been minimally studied in those with ESKD [[13], [14], [15], [16], [17], [18], [19], [20], [21]]. There is also robust evidence that walking can significantly reduce fatigue for adults with chronic illnesses [[22], [23], [24]].

To develop an intervention to meet the needs of those with ESKD, it is necessary to integrate the patient perspective, which can be facilitated through a patient partnership board [[25], [26], [27]]. Patient partnership boards have helped develop interventions for adults with ESKD [[27], [28], [29]]. This study seeks to extend this work by co-developing, with a patient partnership board, a combined progressive muscle relaxation and walking intervention to reduce fatigue in adults with ESKD receiving maintenance hemodialysis. Here we describe this formative work, as well as outline the design and methods of the feasibility trial of the fatigue-reducing intervention.

## 2 Formative work

For our formative work, we worked with a patient partnership board to inform patient-centered adaptation of a fatigue-reducing intervention and to enhance intervention feasibility and relevance for adults with ESKD. The role of the board was to act in a consultative capacity providing feedback, insight, and suggestions on how to adapt the intervention to meet the needs of those with ESKD. Three individuals with ESKD receiving maintenance hemodialysis were recruited via purposive sampling and enrolled as members of a patient partnership board in 2025. The board was kept to three individuals because the board members preferred to participate in meetings via teleconference (Zoom PHI) during hemodialysis, and a smaller board facilitated increased participation in a virtual format while members were receiving hemodialysis [30]. Most meetings were done as 1:1 interviews because board members received hemodialysis at different times. Recruitment was conducted at the University of Illinois Hospital and Health Sciences (UI) Health Dialysis Unit using the following inclusion criteria-being >18 years of age, having a diagnosis of ESKD and receiving hemodialysis for >3 months, able to read and speak English, having fatigue (average over the last week) rated ≥4 via visual analogue scale (0 no fatigue, 10 worst fatigue ever experienced), and being able to stand and walk one block. The exclusion criteria were: the individual's nephrologist refused for them to participate, symptoms of unstable cardiac or pulmonary disease in the last month, lower-extremity amputation without prosthetic, orthopedic or neurologic condition that would preclude walking or tensing/releasing of muscles, uncontrolled orthostatic hypotension, and cognitive impairment that precluded participation. All members of the board provided informed consent, and the University's Institutional Review Board approved the study.

The board was queried about their fatigue and feedback about the intervention components, specifically Bernstein and Borkovec's progressive muscle relaxation [31,32] and a walking intervention to reduce fatigue in adults with cancer [33]. Progressive muscle relaxation is a practice where large muscle groups are tensed and relaxed in a specific order while focusing on the feeling of relaxation. The walking intervention sets individualized step goals tailored to the participant's baseline steps per day and gradually increased based on fatigue levels [33]. The content of the interventions, how they were to be combined, proposed intervention delivery methods, and recruitment and retention methods were brought to the board.

Three 1-h meetings took place utilizing a semi-structured script (Appendix A). During the first two meetings, feedback was sought about fatigue and the proposed intervention. At the third meeting, the refined intervention adapted based on their suggestions was presented. After the third meeting, a 1:1 semi-structured interview (Appendix A) was conducted with each member about the refined intervention. The meetings were recorded, transcribed, and imported into NVivo (2020, QSR International) for analysis. Thematic analysis was conducted by two reviewers (MFH & VD), followed by member checking with two participants who represented each theme. Of note, the purpose of the analysis was to inform patient-centered adaptation and enhance intervention feasibility and relevance, rather than to achieve thematic saturation.

### 2.1 Formative work results

The patient partnership board (n = 3) was comprised of two females and one male, mean age was 65.3 years (range 30-84), and duration of hemodialysis ranged from 2.5 to 7.5 years.

The board described their fatigue, and three themes (Appendix B) emerged from analysis: (1) fatigue is a multifaceted, mind-body symptom (it was both constant and intermittent (having “good and bad days”), it made you not want to do things, and it had a mental and emotional component), (2) there were few things that made fatigue better (moving around, attitude, and time since the last hemodialysis treatment potentially made fatigue better), and (3) receiving hemodialysis and one's health status made fatigue worse (hemodialysis led to fatigue, as did comorbid health conditions and physical inactivity).

After presenting the two interventions, the board was asked what may hinder or promote engagement with the intervention. Three themes (**Appendix C**) emerged for what may hinder engagement: (1) the participants' attitude, mindset, and mood, (2) the participants’ health status (“good and bad days”, symptoms), and (3) progressive muscle relaxation will require practice. Conversely, when asked about what may promote engagement, six themes (**Appendix C**) emerged: (1) doing both progressive muscle relaxation and increasing steps seemed reasonable, (2) individualized, tailored plan for step goals, (3) individualized plan of when participants would do progressive muscle relaxation, (4) interesting in-person training and make it fun to do, (5) positive feedback and encouragement, and (6) adherence monitoring via technology and self-tracking.

Feedback was also sought about the study fitness watch, accelerometer, website, and text messages. For the fitness watch, the board emphasized the importance of ease of use without an excessive number of buttons and a bright screen. The belt-worn accelerometer was described as the easier device to wear, compared to one applied with a bandage. The board shared the importance of the website reiterating information taught in brief, basic, and straightforward ways with pictures or figures and that audio recordings are helpful. For the text messages, the board thought it would be helpful if messages were encouraging and provided positive feedback, as well as reminders that were easy to read. The board found the attention control topic of reading a nutrition label to be helpful, and information about healthy snacks and why some foods should be avoided were suggested as topics of interest. Regarding survey administration format, in-person administration was most appealing, with phone administered also being reasonable but text-delivered being least ideal.

The board was asked about recruitment and retention. The board unanimously agreed that in-person recruitment was ideal, specifically done during hemodialysis with before or after treatment being alternative options. Positive engagement experiences were said to promote engagement and retention, specifically through interactions between the research team and participants. Encouragement and support from the research team and sharing the benefits of participating were also described as promoting retention. Making the study convenient, with study activities primarily taking place during hemodialysis, and surveys not being overly lengthy, were also described as ways to support retention.

The findings from this formative work informed the development of the *Fight Fatigue* intervention and its associated procedures, which we describe below.

## 3 Study protocol methods

### 3.1 Study design & aims

The study is a 12-week pilot feasibility randomized controlled trial. The study aim is to investigate the feasibility and acceptability of the *Fight Fatigue* progressive muscle relaxation and walking intervention in adults with ESKD and fatigue receiving maintenance in-center hemodialysis (n = 40). The trial is registered at clinicaltrials.gov (NCT07014111) and approved by the University of Illinois Chicago (UIC) IRB (protocol #2025-0281).

### 3.2 Study setting & recruitment

Participants will be recruited from UIC, UI Health, and select dialysis facilities with which the study team has relationships. The study team will review electronic medical records to identify those who meet inclusion criteria identifiable via chart review: age ≥18 years, diagnosis of ESKD and receiving hemodialysis for >3 months, and being able to read and speak English; and have none of the chart-identified exclusion criteria: unstable angina, unstable pulmonary disease or pulmonary symptoms that preclude participation, lower-extremity amputation (below or above the knee amputation) without prosthetic, orthopedic or neurologic condition that would preclude walking or tensing/releasing of muscles, cognitive impairment that precludes trial participation, or any other condition that would preclude participation as deemed by the researchers. We will not include patient partnership board members or patients deemed unable to participate, as determined by their nephrologist. Framed by the recommendations of the board, we will recruit participants by approaching them at the dialysis clinic. If they express interest in learning more about the study, a study staff member will provide them with an informational handout and tell them about the study. Additionally, there will be flyers in the waiting room. A passive mailing or emailing strategy may also be used for recruitment at UI Health.

Interested individuals will be screened in person at the dialysis clinic or over the phone, per the individual's preference, to ensure the individual meets inclusion/exclusion criteria after they provide verbal consent to undergo screening. Screening will consist of clarifying any inclusion or exclusion criteria that were not clear on chart review and asking whether they meet the self-reported inclusion criteria, specifically having a fatigue score ≥4 over the last week measured via visual analogue scale (0 being no fatigue and 10 being extreme fatigue), being able to stand and walk one block, and having a cell phone that can receive text messages. Individuals meeting eligibility will be offered the opportunity to complete the consent process and be enrolled.

### 3.3 Randomization

Participants will be randomized after consent and baseline testing are completed. The REDCap randomization module will be designed by the study statistician. Randomization will be stratified based on baseline fatigue scores as measured by the Functional Assessment of Chronic Illness Therapy-Fatigue (FACIT-F) scale (FACIT-F score <30 vs. **≥**30). Randomization within each stratum will be 1:1 to each of the two groups, utilizing a block randomization scheme (block size 4). The study statistician will create this allocation schedule.

### 3.4 Blinding

Due to the nature of behavioral research, blinding will be done to the extent possible, given the nature of this work. The statistician will be blinded to the meaning of the randomization allocation labels (“1” and “2”). The outcomes assessor will be blinded to the extent possible when assessing patient-reported outcomes at weeks 6 and 12 (e.g., fatigue). The research team members delivering the intervention will not be blinded due to the nature of the intervention.

### 3.5 Interventions

***Fight Fatigue*-** The *Fight Fatigue* intervention was informed by two evidence-based interventions (progressive muscle relaxation [31,32] and an increased step count intervention [33]), through our work with a patient partnership board, and guided by the Information-Motivation-Behavioral Skills Model [34]. We combined and refined these interventions to meet the needs and preferences of adults with ESKD, as described above. Refinements suggested by the board were integrated if they aligned with the goal of the intervention (reduce fatigue), maintained the fidelity of the evidence-based interventions, aligned with the guiding theory (Information-Motivation-Behavioral Skills Model), and did not increase participant risk. The intervention delivery is framed by the Information-Motivation-Behavioral Skills Model to support behavior change by providing information to support knowledge, identifying and addressing factors that influence motivation, and assuring that participants have the necessary skills to perform the intervention activities [34].

The *Fight Fatigue* intervention is delivered via two, scripted in-person education/training sessions followed by text messages. During the in-person sessions, participants will learn and practice progressive muscle relaxation and how to increase steps, as well as be provided with written instructions and a place for tracking progressive muscle relaxation times and reaching one's step goal. Progressive muscle relaxation training will begin with a description of the rationale for progressive muscle relaxation, followed by training and demonstration of the tension/relaxation 16-muscle sequence [31,32]. After learning the 16-muscle sequence, the 7- and 4-muscle sequences will be taught [31,32]. Participants will be instructed not to tense/relax muscle groups that are injured or cause pain. After the first training session, participants will be instructed to practice progressive muscle relaxation for 20 min at least 2 days/week. Each participant will receive a unique login to a study-specific website that contains recordings of the progressive muscle relaxation 16-, 7-, and 4-muscle sequences. Progressive muscle relaxation recordings have been used in other studies integrating home-based progressive muscle relaxation [35].

The increasing steps training will consist of learning about taking steps (walking) at light intensity (Borg rating of perceived exertion 2-3). Participants will be given and taught how to use a fitness watch (i.e., Garmin vivosmart). Fitness watches have been used in other interventions aimed at enhancing physical activity among those with ESKD [36]. The fitness watch will be set up by study staff and associated with a deidentified account. Study staff will sync the device throughout the study (approximately every 1-2 weeks). Participants will be given an individualized step goal based on their baseline step count and tailored to their level of fatigue, as has been done in other work [33]. The baseline steps will be determined by one week of wear of the fitness watch (worn with screen not visible to participants). To be included in the calculation of baseline steps, a valid day will be considered to have at least 10 h of wear time, as used in other studies that have utilized consumer wearable technologies in those receiving hemodialysis [37]. Each week, participants will be asked what their fatigue level has been over the last week (rated via visual analogue scale; 0 no fatigue, 10 extreme fatigue). Of note, this weekly fatigue measurement is used for intervention tailoring and is not a primary outcome measure. If a person's fatigue level remains unchanged or improved (from one week to the next), the step goal will be increased, as done by the intervention [33] guiding this component of *Fight Fatigue*. Specifically, if a participant's baseline steps are >7000 steps, the increase would be 0%; if baseline steps are 5001-7000 steps, the increase would be 2.5%; if baseline steps are 2500-5000 steps, the increase would be 5%; and if baseline steps are <2500, the increase would be 7.5%. If fatigue worsens from one week to the next, the step goal for the following week is not increased [33]. Participants will be encouraged to reach their step goal at least two days per week. Participants will be instructed to contact study staff if they have any questions about increasing steps, the fitness watch, and they will be instructed to stop walking if they experience any chest pain, shortness of breath, or otherwise feel unwell.

***Attention Control-*** We will be controlling for contact, not content, in our attention control condition of ESKD Education (information on how to read a food nutrition label). This topic was chosen because it does not influence fatigue and was endorsed by the board as a topic of importance and interest. The attention control condition will be delivered via two, scripted in-person sessions, followed by text messages.

#### 3.5.1 Text messages

After the in-person sessions, all participants will receive text messages for the duration of the study (approximately 4 text messages per week) that reinforce information discussed. The messages are the same for each participant (based on group assignment), sent in the same order, and participants are asked to reply back “1” after reading. The messages are framed by the Information-Motivation-Behavioral Skills Model, specifically providing information, addressing factors that influence motivation, and reinforcing skills [34]. Approximately every 1-2 weeks, study staff will verify with each participant that they are receiving text messages.

### 3.6 Research staff training & fidelity

Training will be provided for research team members and refreshed every 3 months. The in-person sessions are scripted to enhance fidelity. The PI will routinely observe research staff conducting sessions for fidelity.

### 3.7 Retention

To promote retention, we will schedule meetings with participants at times convenient for them, including while receiving hemodialysis. Participants receive incentives for completing the baseline and end of study visits, as well as a fitness watch (e.g., Garmin) at end of study (intervention participants will keep the one they received during the intervention; attention control participants will receive a new fitness watch), and if they participate in an optional 1:1 interview (intervention participants only).

### 3.8 Study endpoints

Data will be collected at baseline, week 6, and end of study (week 12). Baseline and end of study assessments will be done in-person. The week 6 assessment will be either by phone or in person.

#### 3.8.1 Primary endpoint

The primary study endpoints are feasibility and acceptability. We will examine recruitment, enrollment, and attrition metrics. We will measure adherence to the intervention, defined as participating in at least two progressive muscle relaxation sessions per week, which will be evaluated via individual login accesses and duration of each access to the study website, and having at least two days per week where they met their step goal, evaluated via the fitness watch. We will evaluate adherence to the text messages by evaluating number of messages read compared to the number of messages sent. We will also evaluate acceptability via Likert scale, visual analogue scale, and open-ended questions. Additionally, intervention participants will have the option to participate in an optional 1:1 interview to further explore acceptability of the intervention at the end of the study.

#### 3.8.2 Secondary and exploratory endpoints

The secondary endpoint is fatigue, quantified by the FACIT-Fatigue survey, which assesses 7-day fatigue and is a recommended measure for adults with ESKD [4,38]. FACIT-Fatigue will be evaluated at baseline, week 6, and end of study. Fatiguability will be evaluated with the Pittsburgh Fatiguability Scale, a survey of expected physical and mental tiredness related to various activities [39]. Self-reported sleep quality will be measured with the PROMIS Sleep questionnaire [40]. Pain will be assessed using the PROMIS Pain Intensity and Interference questionnaires [41]. The SF-36 will be used to evaluate self-reported quality of life [42]. Self-efficacy for physical activity will be evaluated using a scale assessing readiness for change to increase physical activity [43]. Given the influence of patient activation on behavior [44,45], the Patient Activation Measure (PAM-13) will be evaluated [46,47]. Since the board described fatigue as a mind-body symptom, we will examine the Whole Person Health Index (WPHI), a self-assessed holistic view of one's health [48].

In addition to questionnaires, we will evaluate daily physical activity and inactivity (e.g., METs, steps, sedentary time, sleep, etc.) as well as sleep quality via accelerometer at baseline and end of study with 1 week wear of the ActiGraph (GT3X), a validated measure of activity and sleep quality that has been used with adults with ESKD [[49], [50], [51]]. Participants will fill out a log while wearing the ActiGraph to report sleep and non-wear times. A minimum of 5 valid days of wear will be included, and a valid day will be considered at least 8 h of wear time, which has been established as an acceptable parameter for reliability [52]. The sampling rate of the devices will be 30 Hz (Hz). For analysis, epoch length will be set to 10 s. Bout parameters and METs will be evaluated using the Freedson (1998) and Freedson EE algorithms [53]. As per other work using the ActiGraph in adults with ESKD, the cut points for analysis will be defined as < 100 counts/min for sedentary time, 100 to 1951 counts/min for light physical activity, and ≥1952 counts/min for moderate-to-vigorous physical activity (MVPA) [54].

At baseline and end of study, we will evaluate parameters collected as part of routine clinical dialysis care, specifically Kt/V (dialysis clearance), body mass index, dialysis fluid removal, number of missed dialysis treatments, blood pressure, heart rate, dry weight, hemoglobin level, albumin level, phosphorus level, and medications.

### 3.9 Data analysis

The study hypothesis is that the recruitment ratio (percent of approached individuals who agree to participate) will be >60%, retention (percent of randomized individuals who complete the final 12-week study assessment) will be >80%, and adherence among retained participants (at least two progressive muscle relaxation sessions/step goal achieved per week; text messages read) will be >70%. We hypothesize that greater than 80% of retained participants will rate the study as acceptable (rating of agree or higher on end of study acceptability questions). Feasibility metrics and baseline descriptive statistics will be summarized. We will evaluate acceptability via Likert-scale, visual analogue scale, and open-ended questions. Mean scores will be calculated, and scores in the range from disagree to strongly disagree will be considered unacceptable conditions. Open-ended question answers will be summarized by the PI, and the summary will be reviewed by a research team member to verify accuracy. Adherence will be evaluated via fitness watch (steps) and number/duration of accesses on the website. Additionally, steps will be evaluated via accelerometer. Text message adherence will be assessed based on messages sent and read.

We will then estimate within group variability of change in the secondary outcome measure (FACIT-Fatigue) to appropriately power the future, fully powered trial informed by this feasibility trial. We will estimate correlation structure between the secondary outcome measure and Kt/V, dialysis fluid removal, dialysis dose, blood pressure and heart rate, change in blood pressure during treatment, number of missed dialysis treatments, medications, body mass index, weight, hemoglobin, albumin, and phosphorus to appropriately assess power for the future, full powered trial.

For missing data, we will use a complete case approach, as our preliminary work suggests low rates (<1%) of missing data. Multiple imputation methods will be used as sensitivity analyses to ensure robustness of findings if there is higher missingness than anticipated. We will monitor missing data and identify predictors of missingness, specifically evaluating demographic factors, including but not limited to age and sex, as predictors of missingness, and examine the acceptability of outcome measures to inform the future efficacy trial.

#### 3.9.1 Sample size

The sample size will consist of 40 participants, and we anticipate an attrition rate of no greater than 20% based on our team's previous work. This sample size will allow us to assess both feasibility and acceptability to a reasonable level of precision (estimating retention and intervention completion rates within a maximum range of error of 14%), achieve the necessary estimates to design a future, well-powered interventional trial, and it falls within well accepted guidelines on sample sizes for pilot studies of this type [55,56]. We will conduct Intention to Treat and Per Protocol Analyses [57].

## 4 Discussion

This trial aims to determine the feasibility of a fatigue-reducing intervention that combines progressive muscle relaxation and increasing steps for adults with ESKD receiving in-center maintenance hemodialysis. This trial is particularly relevant given the pervasiveness of fatigue in those with ESKD and the paucity of patient-centered, low-demand interventions to address this symptom. Per our review, our study is among the first to evaluate a combined mind-body intervention in adults with ESKD, co-developed with the support of a patient partnership board. Our study utilizes progressive muscle relaxation, which can reduce fatigue for other chronic disease populations but has been minimally explored in those with ESKD [[17], [18], [19],^21^,^22^,^58^,^59^]. The increasing steps component is novel in its utilization of tailored step goals based on fatigue and baseline steps [33,36,60].

Our work with the patient partnership board helped to characterize the experience of fatigue, which informed intervention development. Our findings contribute to understanding the lived experience of fatigue for those with ESKD. Fatigue has been described by those with ESKD in other studies as abnormal and excessive tiredness that may not improve with rest and can make daily activities difficult [61,62]. Our work highlights that fatigue is a mind-body experience, which has been described in other studies in the bidirectional relationship between mood and fatigue for those with ESKD [61]. The board informed the development of our intervention and provided insight into factors influencing participation. There has been minimal exploration into the perspectives of those with ESKD about mind-body interventions, and our work adds to findings from previous work on patient perspectives about physical activity interventions for those with ESKD [63].

Our study was not without limitations. Our work with the board reflects the experience of three individuals, so the findings may not be representative of all those with ESKD. We hope to begin to address this in the optional post-intervention 1:1 interviews done with intervention participants. The board was recruited from a single dialysis center, which may not reflect all patients’ experiences and therefore also limits the representativeness of the findings. The board shared feedback on the intervention, but did not participate in it, so their recommendations may not reflect the perspective of individuals taking part in the intervention. Most board meetings were conducted as 1:1 interviews, which may have limited mutual sharing of ideas. The purpose of the proposed trial is to examine the feasibility of a combined mind-body intervention. We acknowledge the limitation that this feasibility trial is not intended to determine efficacy of the combined intervention or the efficacy of the individual effects of each intervention component. We will seek to address this in the subsequent efficacy trial informed by this work that will examine the individual and combined intervention components. For the trial protocol, the attention control condition of nutrition label education was chosen because it was a topic of importance for this patient population and was emphasized as a topic of interest by the board. We acknowledge that there is a remote possibility that nutrition label education could change dietary habits that potentially have a role in fatigue, but we believe this possibility is minimal. The trial protocol will utilize blinding only to the extent possible in a behavioral intervention trial, which does not allow for full blinding. We will blind as much as possible in the protocol, specifically the outcomes assessor will be blinded to group during the week 6 and end of study assessments of patient-reported outcomes (e.g., fatigue).

There has been minimal investigation into the feasibility and acceptability of multicomponent mind-body interventions to reduce fatigue in ESKD. Our work with a patient partnership board to formulate this feasibility trial is a first step. The findings of the described feasibility trial will provide information to develop a larger-scale fatigue-reducing intervention for adults with ESKD receiving hemodialysis.

## CRediT authorship contribution statement

**Mary F. Hannan:** Writing – review & editing, Writing – original draft, Resources, Project administration, Methodology, Investigation, Funding acquisition, Formal analysis, Data curation, Conceptualization. **Michael J. Fischer:** Writing – review & editing, Methodology, Investigation, Conceptualization. **Victoria deMartelly:** Writing – review & editing, Formal analysis, Data curation. **Natalie Lif:** Writing – review & editing, Data curation. **Nathan Tintle:** Writing – review & editing, Methodology, Investigation, Conceptualization. **Ardith Z. Doorenbos:** Writing – review & editing, Supervision, Methodology, Investigation, Conceptualization.

## Funding for this study

Research reported in this publication was supported by the National Center for Complementary and Integrative Health R34AT012770. The content is solely the responsibility of the authors and does not necessarily represent the official views of the National Institutes of Health.

The authors report no conflicts of interest.

## Declaration of competing interest

The authors declare that they have no known competing financial interests or personal relationships that could have appeared to influence the work reported in this paper.

## Data Availability

No data was used for the research described in the article.
