# Supplementary material for: A combined progressive muscle relaxation and walking intervention for adults with end-stage kidney disease receiving hemodialysis (Fight Fatigue): Study development and protocol
Source: Contemp Clin Trials Commun. 2026 Mar 24;51:101631. doi: 10.1016/j.conctc.2026.101631 (PMC13054067; doi:10.1016/j.conctc.2026.101631)
Supplement: Multimedia component 1 [file mmc1.pdf]

Appendix A- Patient Partnership Board focus groups/interview script & Final interview script

<u>Examples of questions- Meetings #1-3</u>

What are your thoughts about fatigue?

How would you describe the feeling of fatigue that you experience? What does it feel like for you?

What are some things that influence your fatigue? Like things that make the fatigue worse or better?

What do you think about this relaxation technique?

What are your thoughts on what might make doing this relaxation technique difficult for people receiving dialysis?

What do you think might make this relaxation technique more appealing?

What do you think about having people do the relaxation technique for 20min during the middle part of a dialysis session, where a research staff member may be there to answer any questions?

What do you think about the in person plan for instructions and training on this relaxation technique?

What do you think about having the website available for people? Is there anything you would want to see on the website?

If you were in the study, do you think you would want to do the relaxation during dialysis or a time when you are at home? And why?

If you were in the study and doing the relaxation at home, do you think it would be helpful to have audio recordings of the instructions of how to do the relaxation? And why?

What do you think about this steps program?

What are your thoughts on what might make doing this steps program difficult for someone receiving dialysis?

What do you think might make this steps program more appealing?

Is there anything that, if you were doing this part of the intervention, you would want to hear about or learn about increasing steps or the fitness watch?

What do you think about having our research team check in with people at the dialysis unit, after the training is complete, to see how the relaxation is going and be available for questions?

What do you think about having people keep track on their own of how many times they do the relaxation in a log where they write down when and for how long they do the relaxation?

What do you think about if we would send a text message that would have a link to a survey that people would fill out?

What do you think about people being asked survey questions in person here at the dialysis unit?

When do you think that people would want to be approached to hear about our study?

How do you think that people would want to be approached to hear about our study?

<u>1:1 Interview question examples</u>

On a scale from 0 to 10 (0 being the worst, 10 being the best), what rating would you give the refined intervention and why?

Would you recommend this intervention to someone you know who receives dialysis? And why or why not?

What could be done to make this intervention better meet your needs or the needs of others receiving dialysis?

What could be done to make the intervention more appealing?

Is there any other feedback you would like to share about the intervention itself, recruitment for the intervention, and ways to encourage people staying engaged with the study for the 12-week duration of the study?

Any other feedback about the fitness watch we chose that you would like to share?

And lastly, we started our first meeting in March talking about fatigue. Is there anything else about fatigue or your experience of fatigue that you would like to share with us?

Appendix B- Qualitative Analysis Themes- Thoughts on Fatigue

Thoughts on fatigue	
**Fatigue is a multifaceted, mind-body symptom**	
Constant symptom	I feel that sometimes it's hard to deal with ... I don't like feeling tired all the time. (Participant A)
Challenging symptom	I don't know what else to say except that it's really annoying. (Participant A)
Relates to how you feel emotionally and your mood	I think fatigue is just something that happens to you when you're really down. (Participant A)<br><br>It probably has to do a lot with my mood, you know, how I'm doing…(Participant C)
Get used to it	I mean, thankfully… I've been able to get used to it… (Participant C)
Good and bad days	…some days I have good days, some days I have bad days. (Participant C)
Makes you not want to do things	Fatigue is…it makes you feel tired…Not energetic. It makes you feel like you want to do things, but you can't... (Participant A)
Mental aspect of fatigue	… I believe the fatigue is a more… a mental problem than a physical problem. (Participant C)
Painful	I mean, I feel tired and I feel, I just feel bad… like my head hurts. (Participant C)
Tired and want to sleep but sleep may not help	I just feel tired. That's it…it's like a tired that you can't get rid of even if you sleep. (Participant C)
**What makes fatigue better**	
Attitude	…the mindset of how you're dealing with your dialysis directly correlates to your…fatigue. (Participant C)
Family	I think you need people, you need family, and that helps. (Participant A)
The further out from dialysis you are	It's just something that just goes away …with time…if I have dialysis in the morning, I'll feel better around 5 or 6, regardless if I sleep or not. (Participant C)
Moving around and doing things	It's just the moving around… your body just moving, doing something... (Participant B)
Nothing	My experience so far has been just, I just feel tired. That's it…it's like a tired that you can't get rid of even if you sleep. (Participant C)
Visualizing happy things	… I try to visualize some things that made me happy. (Participant A)
**What makes fatigue worse**	
Dialysis	I guess with … cleaning your blood and everything… it takes a lot out of you. So you always feel very run down. (Participant B)

Physical inactivity	… everyone wants to wait until they're not tired to do things. And I feel like that pushes their fatigue more because as you're waiting, you're not doing anything, so …your body gets used to not doing anything. And so now your fatigue increases because now … the things that you used to do make you even more tired. (Participant C)
Other comorbidities	… with me, with [*medical condition*], moving around a lot, you know, will cause a fatigue… with me too. (Participant B)

Appendix C- Qualitative Analysis Themes- Feedback on the Intervention

Feedback on intervention overall and the individual components	
**Factors that may make intervention engagement difficult**	
Attitude, mindset, and mood	
Mindset to do progressive muscle relaxation during dialysis	It all depends on how they come in and how they're feeling... Because a lot of times when you come to dialysis, it's just enough to get through dialysis … It's just enough to get to dialysis. (Participant B)
Mindset will impact ability to reach step goal	When they're in a…depressed state might not even want to do anything ... (Participant C)<br><br>… we don't so much concentrate on how many steps we're taken. (Participant B)
Health status	
How you feel	… sometimes I'm in pain. And it would be hard to motivate myself to do that when I'm feeling that achy. (Participant A)<br><br>… if it's a good day or not. You know, every day is not a good day. (Participant B)
Impact of health status	I think it depends how you're feeling that day... Sometimes you're more energetic than others. (Participant A)<br><br>I think because you move and … you're getting more active, I think you feel better inside. (Participant A)
Progressive muscle relaxation will require practice	..the whole relaxation thing… it sounds to me like it's a technique that you need to kind of get good at…when something needs practice, that means you're not going to see the full benefits until you are finishing whatever you're doing. (Participant C)
**Factors that may promote engagement with the intervention**	

Doing both components seems reasonable	… they'd be a good complement to each other…relax when you have to relax, but move when you've got to move. (Participant C)<br><br>I would look forward to having something to do… knowing that I had something to do... (Participant B)
Encouragement from others and interacting with staff	…you have to more or less …kind of … encourage people... (Participant B)
Other people and encouragement can support motivation	Sometimes it works if you have… another person that's working along with you. (Participant B)
Feedback on doing relaxation with staff present in person	I think…in person … would probably be the best way to really teach, and not only teach, but motivate people…to actually try it. (Participant C)
Feedback on in person training	I think it's… helpful because when you see somebody do something and you're doing it, and it's in person, if you're doing it wrong, they could tell you, instead of you doing it on your own …it's good to have somebody guide you. (Participant C)
Individualized plan	
Finding the time to do steps may be hard	…steps are good and you can do it throughout the day, but it also takes time…that's probably one of the things I lack the most... (Participant C)
Individualized plan for doing progressive muscle relaxation	
Concerns doing during dialysis	
Comorbidities and symptoms may make progressive muscle relaxation difficult	It’s just enough to get to dialysis. (Participant B)<br><br>Because this is exhausting. You know, taking dialysis is exhausting. (Participant B)

Dialysis access or blood pressure cuff related limitations	And I probably wouldn't have much to worry about setting off the machine, unless they're taking the blood pressure at the time. (Participant A)
Dialysis is too stressful for progressive muscle relaxation	I would probably do it more at home, because it would be more of a restful place. (Participant A)
Limited space while on dialysis	…doing it while during dialysis…it might be hard because you're sitting in one position… (Participant B)
Problem if it causes cramping	I think it's just that people might… accidentally cause… cramps and stuff like that… (Participant C)
Timing doing progressive muscle relaxation during dialysis	I think… middle of the treatment is your best bet. If you wait… too long… that's when… most of the liquid has been taken off, and that's when the cramping usually starts. (Participant C)
Progressive muscle relaxation better to do at home	I think doing it at home would be more relaxing because you'd be in your own space and probably your own recliner, your own bed. (Participant B)
Progressive muscle relaxation better to do during dialysis	I mean, here [*at dialysis*] I'm not doing anything, so I guess it's easier. But I feel like at home I'm already more relaxed. Here [*at dialysis*], I'm not. (Participant C)
Individualized plan for reaching step goal	

Ideas of ways to increase steps	… because on dialysis days… you're more fatigued than you are on non-dialysis days… (Participant B)<br><br>Um, I think it's [*tailored step goal*] a good idea…especially the having it on a sliding scale. (Participant B)
Just need to try it	… I think just by relaxing and trying it, and giving it a try probably would be good. Instead of…thinking you can't do it, just trying. (Participant A)
Good if it works	I think if it works, it's probably a good thing. I would try it. (Participant A)
Make it easy	And if it's easy to do. (Participant B)
Make the intervention engaging and fun	You know, making it fun…making it so it's something that, you know, that you enjoy doing…It's something that… makes you feel good. (Participant B)
Make learning fun, exciting, and interesting	Make it where it…looks like it'll be fine. Where'll it'll be fun. Where they'll have fun doing it. (Participant B)
Tracking adherence to intervention components feedback	
Feedback on method to track steps	
Consumer-wearable tracked	*Interviewer- Okay, okay. Um, so to keep track of how many steps people are taking, we will meet with them about once per week at the dialysis unit and connect their fitness watch to our computer to tell us how many steps they have been taking. What do you think about that?* That's pretty good. (Participant A)
Self-tracking	…it'd be a good way to increase mindfulness. Like, "Oh, wow, I walked this many steps," or, "Oh, wow, this is all I walked?" … (Participant C)
Progressive muscle relaxation tracking feedback	
Manual or verbal tracking	…it'd be a pretty good idea to help with engagement…a little homework. (Participant C)
Website for tracking	I'd say … with people… it's easier for it to be done for them than to have to do it themselves. (Participant C)
